# Maternal Cigarette Smoke Exposure Worsens Neurological Outcomes in Adolescent Offspring with Hypoxic-Ischemic Injury

**DOI:** 10.3389/fnmol.2017.00306

**Published:** 2017-09-26

**Authors:** Yik L. Chan, Sonia Saad, Rita Machaalani, Brian G. Oliver, Bryce Vissel, Carol Pollock, Nicole M. Jones, Hui Chen

**Affiliations:** ^1^School of Life Sciences, Faculty of Science, University of Technology Sydney, Sydney, NSW, Australia; ^2^Respiratory Cellular and Molecular Biology, Woolcock Institute of Medical Research, University of Sydney, Sydney, NSW, Australia; ^3^Renal Research Group, Kolling Institute, Royal North Shore Hospital, St. Leonards, NSW, Australia; ^4^Department of Medicine, University of Sydney, Sydney, NSW, Australia; ^5^Centre for Neuroscience and Regenerative Medicine, Faculty of Science, University of Technology Sydney, Sydney, NSW, Australia; ^6^Department of Pharmacology, School of Medical Sciences, University of New South Wales, Sydney, NSW, Australia

**Keywords:** mitophagy, apoptosis, cognition, motor behavior

## Abstract

Hypoxic-ischemic (HI) encephalopathy occurs in approximately 6 per 1000 term newborns leading to devastating neurological consequences, such as cerebral palsy and seizures. Maternal smoking is one of the prominent risk factors contributing to HI injury. Mitochondrial integrity plays a critical role in neural injury and repair during HI. We previously showed that maternal cigarette smoke exposure (SE) can reduce brain mitochondrial fission and autophagosome markers in male offspring. This was accompanied by increased brain cell apoptosis (active caspase-3) and DNA fragmentation (TUNEL staining). Here, we aimed to investigate whether maternal SE leads to more severe neurological damage after HI brain injury in male offspring. Female BALB/c mice (8 weeks) were exposed to cigarette smoke prior to mating, during gestation, and lactation. At postnatal day 10, half of the pups from each litter underwent left carotid artery occlusion, followed by exposure to 8% oxygen (92% nitrogen). At postnatal day 40–44, maternal SE reduced grip strength in grip traction and foot fault tests, which were also reduced by HI injury to similar levels regardless of the maternal group. Limb coordination was impaired by maternal SE which was not worsened by HI injury. Maternal SE increased anxiety level in the offspring, which was normalized by HI injury. Apoptosis markers were increased in different brain regions by maternal SE, with the cortex having further increased TUNEL by HI injury, along with increased markers of inflammation and mitophagy. We conclude that maternal SE can worsen HI-induced cellular damage in male offspring well into adolescence.

## Introduction

Oxygen deprivation before and around the time of birth can result in hypoxic-ischemic (HI) brain damage. In humans, during HI encephalopathy, there is a decrease in blood oxygen saturation and blood flow, interrupting normal fetal brain development (Li et al., 2012a). The cerebral cortex, hippocampus, and sub-ventricular regions are the brain regions most vulnerable to HI damage in rats (Northington et al., 2001). Earlier studies showed that maternal smoking causes hypoxia in the fetus of rhesus monkeys with the mechanisms not completely understood (Socol et al., 1982). It has been proposed that nicotine in cigarette smoke can reduce blood flow to the placenta due to its vasoconstrictor properties (Philipp et al., 1984) and that it does so through regulation of specific nicotinic acetylcholine receptor subunits (Machaalani et al., 2014). Human studies also showed that maternal cigarette smoke exposure (SE) can increase the risk of adverse perinatal outcomes, such as compromised brain development of infants due to maternal anemia and fetal hypoxia (Habek et al., 2002). In addition, cigarette smoking can increase carboxyhemoglobin levels during pregnancy which can reduce the oxygen carrying capacity of both fetal and maternal red blood cells (Cole et al., 1972; Bureau et al., 1983).

Normal brain function requires a large amount of oxygen for mitochondrial oxidative phosphorylation (OXPHOS) to produce ATP. HI encephalopathy can rapidly reduce ATP production which is crucial for brain development during the fetal period, due to the impairment of cerebral blood flow and oxygen delivery to the brain in humans (Cotten and Shankaran, 2010). The need to maintain cellular homeostasis after brain injury also increases the demand for ATP synthesis, which increase the byproducts of OXPHOS and free radicals that directly damage the mitochondria. Damaged mitochondria are repaired through a process known as mitophagy (autophagy specific to mitochondria). Mitophagy plays a key role in mitochondrial turnover and tissue repair during brain injury to ensure sufficient energy supply via fission and fusion processes. Fission is separating the damaged mitochondrial fragment from the healthy part of the mitochondria facilitated by several proteins, such as dynamin-related protein-1 (Drp-1), fission-1 protein, phosphatase and tensin homolog-induced putative kinase-1 (Pink-1), and Parkin. Fusion is combining two healthy mitochondrial fragments to form a new mitochondrion via optic atrophy-1 (Opa-1). Indeed, following HI injury in neonatal rat brain, markers of autophagosome [e.g., light chain 3 microtubule-associated protein A/B (LC3A/B)] were increased, suggesting increased autophagy and mitophagy activities (Rocha-Ferreira and Hristova, 2016).

Smoking 10 or more cigarettes per day during pregnancy has been shown to increase the risk of cerebral palsy in the offspring which is a well-known outcome of HI encephalopathy (Streja et al., 2013). Using a mouse model of maternal cigarette SE, we previously showed that brain markers of mitochondrial fission and autophagy in adult SE offspring, were accompanied by reduced levels of mitochondrial antioxidant manganese superoxide dismutase (MnSOD) suggesting increased oxidative stress (Chan et al., 2017). We also found that maternal SE increased markers of hypoxia, oxidative damage and DNA damage (TUNEL), as well as increased markers of apoptosis (caspase-3) in the brains of the SE offspring (Chan et al., 2016b). Male offspring were more vulnerable to the impact of maternal SE than females, reflecting a gender difference (Chan et al., 2016a). Thus, maternal SE may damage brain cells in male offspring into adolescence, via increased oxidative stress in response to an hypoxic intrauterine environment and subsequently impaired mitochondrial integrity in the brain (Chan et al., 2017). This led to our hypothesis that adolescent offspring from the SE mothers would undergo more severe tissue injury and neurological deficits if they experienced a HI injury early in life.

Perinatal nicotine exposure has been shown to enhance the vulnerability to HI brain injury in neonatal rats (Li et al., 2012b). Insufficient removal of damaged mitochondria or excessive degradation of mitochondria can increase cell death during cerebral ischemia (Baek et al., 2014; Shi et al., 2014; Tang et al., 2016). Human studies have correlated maternal SE to impaired locomotor function in young offspring, impaired memory and learning abilities, as well as behavioral problems in adolescent offspring (Orlebeke et al., 1999; Trasti et al., 1999; Huizink and Mulder, 2006; Jacobsen et al., 2006; Lambe et al., 2006). Therefore, we hypothesized that maternal SE would exacerbate cellular injuries and worsen the neurological outcomes in the adolescent offspring following HI brain injury. This study aimed to investigate the impact of maternal SE on brain tissue injury, motor and cognitive functional outcomes (assessed by novel objective recognition, grip traction, foot fault, and elevated plus maze tests), and brain markers of mitophagy, autophagy, and inflammation in male adolescent offspring. HI injury was induced on postnatal day (P) 10 in male offspring and behavioral outcomes were examined at P40–44.

## Materials and Methods

### Animals

The animal experiments were approved by Animal Care and Ethics Committee at the University of Technology Sydney (ACEC# 2014-029). All protocols were performed according to the Australian National Health and Medical Research Council Guide for the Care and Use of Laboratory Animals. Thirty virgin female BALB/c mice (6 weeks, Animal Resources Centre, Perth, Australia) were housed at 20 ± 2°C and maintained on a 12-h light, 12-h dark cycle (lights on at 06:00 h) with *ad libitum* access to standard laboratory chow and drinking water. Maternal SE and breeding were performed as previously described (Chan et al., 2016b, 2017; Tang et al., 2016; Vivekanandarajah et al., 2016). In brief, the female dams were subjected to whole body exposure to the smoke produced by two cigarettes (Winfield Red, ≤16 mg tar, ≤1.2 mg nicotine, and ≤15 mg of CO; VIC, Australia) in a perspex chamber (19L), twice daily from 6 weeks prior to mating, during gestation, and lactation. SHAM dams were exposed to normal air in an identical chamber for same period of time.

At P10, half of the pups from each litter were anesthetized by 2.5% isoflurane (1% O_2_, Veterinary Companies of Australia, NSW, Australia) and underwent left carotid artery occlusion as we have previously published (Jones and Bergeron, 2001). This procedure was undertaken in P10 mouse pups given the brain of the mice at this time point is equivalent to that in the human neonate (Dutta and Sengupta, 2016). Briefly, an incision was made on the left side of the neck. The tissues and fat were teased carefully to expose the left carotid artery for ligation. The sham surgery was performed without carotid artery ligation. The wound was closed by Vetbond^TM^ glue (3M, MN, United States).

Sixty minutes after the surgery, the pups were exposed to 8% oxygen (92% nitrogen) in a humidified chamber for 30 min in a 37°C water bath to induce HI injury. The other half of the litter underwent sham surgery and were exposed to room air under the same conditions. This resulted in four experimental groups (SHAM: air-exposed dam with sham surgery; HI: air-exposed dam with HI injury; SE: cigarette smoke-exposed dam with sham surgery; SEHI: cigarette smoke-exposed dam with HI injury; *n* = 12). The brains of the male pups were harvested at P45. A portion of the brain (bregma -1 to 4 mm) was snap frozen for real-time PCR and western blotting analysis. A portion of the brain (bregma -1 to -2 mm) was fixed in 10% formalin for histology or immunohistochemistry.

### Behavioral Tests at P40–44

Behavioral tests were performed on P40–44 which is equivalent to adolescence stage in humans (Dutta and Sengupta, 2016).

#### Novel Objective Recognition Test

This test evaluates short-term memory retention. Each mouse was placed in a dark-colored box containing two identical green square blocks for two 5-min sessions, familiarization and test phases (5 min interval) as we have published (Chen et al., 2016). During the test phase, one of the objects was replaced with an orange triangular shaped object. The time spent exploring each object was recorded as previously published. The results are presented as the percentage of the total time spent with the new object out of the total time spent with both objects as previously published (Quinn et al., 2008; Thanos et al., 2015). It is the nature of a mouse to explore a novel object over a familiar one. A mouse with a cognitive deficit will not be able to remember the old object during the test phase, therefore will spend a similar amount of time with each object.

#### Grip Traction Test

Forelimb muscle strength was tested by the ability of a mouse to hang on to a plastic rod (0.6 cm in diameter, 50 cm above the ground horizontally) by the front limbs as previously published (Jones et al., 2008). The test lasted a maximum of 2 min and was stopped when the mouse fell off or when the hind limbs were placed onto the rod. A foam pad was laid under the rod to prevent the injury due to the fall.

#### Foot Fault Test

This tests the motor coordination function. The mouse was placed on a horizontal grid (20 cm × 20 cm, square 1 × 1 cm). When a mouse misplaced a forelimb or hindlimb, the foot fell into the grid squares. The number of foot faults and total number of footsteps taken within 2 min were recorded. The results were expressed as the percentage of foot faults out of the total number of steps taken.

#### Elevated plus Maze

This tests the anxiety. Each mouse was placed in the cross section of the elevated plus maze for 10 min as previously published (Komada et al., 2008). The time spent in the closed and open arms was recorded. The mouse will spend more time in the closed arms if it is anxious. The results are expressed as the percentage of time spent in the two open arms out of total time spent on both open and closed arms.

### Western Blotting

The protein levels of mitophagy fission markers (Drp-1, fission-1 protein, Pink-1, Parkin), fusion marker (Opa-1), autophagy marker (LC3A/B-I and II), endogenous antioxidant (MnSOD), and mitochondrial functional markers [translocase of outer membrane (Tom)-20, Tom-40, and OXPHOS complexes I–V] were measured by western blotting. The brain hemisphere ipsilateral to the occluded carotid artery was homogenized using lysis buffer for whole protein and mitochondrial protein extraction as described and previously published (Chan et al., 2016b). As such, the data presented is for the whole brain hemisphere. Protein samples (20 μg) were separated using NuPage Novex 4–12% Bis-Tris gels (Life Technologies, CA, United States) and transferred to PVDF membranes (Rockford, IL, United States), which were blocked with non-fat milk and incubated with primary antibodies. Samples were incubated with primary antibodies [Drp-1 (1:2000, Novus Biotechnology, United States), Fission-1 (1:500, Santa Cruz Biotechnology, United States), Pink-1 (1:2000, BioVision Incorporated, United States), Parkin (1:500, Cell Signaling Technology, United States), Opa-1 (1:2000, Novus Biotechnology, United States), LC3A/B (1:2000, Cell Signaling Technology, United States), MnSOD (1:2000, Millipore, United States), Tom-20 (1:2000, Santa Cruz Biotechnology, United States), Tom-40 (1:2000, Santa Cruz Biotechnology, United States), OXPHOS complexes (1:2500, Abcam, United Kingdom)] overnight at 4°C, and appropriate secondary antibodies (goat anti-rabbit or rabbit anti-mouse IgG horseradish peroxidase-conjugated secondary antibodies. Protein expression was detected by SuperSignal West Pico Chemiluminescent substrate (Thermo Fisher Scientific, CA, United States) and Fujifilm LAS-3000 (Fujifilm, Tokyo, Japan). The density of the protein bands was determined using ImageJ software (National Institutes of Health, Bethesda, MD, United States). The results are expressed as a ratio of the intensity of the protein of interest relative to the band intensity of β-actin or cytochrome c oxidase subunit (COX) IV.

### Real-Time PCR

Total mRNA was extracted from ipsilateral brain tissues using Trizol reagent (Thermo Fisher Scientific, CA, United States). Regional comparisons (between cortex, hippocampus, and hypothalamus) were not possible due to low amount of tissue and as such, the data presented is for the whole brain hemisphere. The purified total RNA was used as a template to generate first-strand cDNA using M-MLV Reverse Transcriptase, RNase H, Point Mutant Kit (Promega, Madison, WI, United States) as described in our previous study (Chan et al., 2016b). Genes of interest were measured using manufacturer pre-optimized and validated TaqMan^®^ primers and probes (Thermo Fisher Scientific, CA, United States). The probe sequence of the inflammatory markers tested provided by the manufacturer are as follows (IL-1β, probe TCCTTGTGCAAGTGTCTGAAGCAGC, NCBI references: NM_008361.3, M15131.1, BC011437.1, ID Mm01336189_m1; IL-6, probe ATGAGAAAAGAGTTGTGCAATGGCA, NCBI references: NM_031168.1, X06203.1, X54542.1, ID Mm00446190_m1; iNOS, probe GGCCTTGTGTCAGCCCTCAGAGTAC, NCBI references: NM_010927.3, ID Mm00440502_m1). The probes of the target genes were labeled with FAM and those for housekeeping 18s rRNA were labeled with VIC. Gene expression was standardized to 18s RNA. The average expression of the control group was assigned as the calibrator, against which all other samples are expressed as fold differences.

### Immunohistochemistry

Brain morphology, apoptosis, and DNA damage were assessed in the formalin fixed paraffin embedded sections using hematoxylin and eosin staining, active caspase-3 and TUNEL staining (*n* = 5). The advantage of the staining was that it allowed for regional differentiation amongst the cortex, hippocampus, and hypothalamus. For all staining, the sections were placed in xylene, and then hydrated by being taken through gradient ethanols with reduced concentrations to distilled water.

For hematoxylin and eosin staining, the slides were stained with Mayer’s hematoxylin and eosin staining after hydration. The size difference between the left and right hemispheres from each animal was measured with ImageJ (National Institutes of Health, Bethesda, MD, United States). Three sections that were 70 μm apart at ∼bregma -1 mm were used to measure the injury size.

For active caspase-3 and TUNEL staining, the sections underwent heat-induced epitope retrieval by microwaving (Homemaker; EM926ENV;900W) for 17 min in 10% Tris-EDTA antigen retrieval buffer (1 mM EDTA, 1 mM sodium citrate, 2 mM Tris, pH 9.0) followed by cooling in a water bath for 15 min.

For active caspase-3 staining, the tissues were incubated with active caspase-3 antibody (1:300 dilution using 1% normal horse serum, catalog no. 559565, BD Biosciences, Australia) overnight at room temperature. Negative controls were incubated with 1% normal horse serum only. The sections were then incubated with a secondary antibody (biotinylated anti-rabbit made in horse, 1:200, BA-1100, Vector Laboratories Inc., United States) for 45 min at room temperature, followed by avidin–biotin peroxidase reagent (VEPK4000, Vectastain ABC kit; Vector Laboratories, United States) for 40 min and then visualized using the diaminobenzidine (DAB) chromogen for 8 min. Sections were subsequently counterstained with hematoxylin and coverslipped.

ApopTag^®^ Peroxidase kit (S7100, Merck Millipore, VIC, Australia) was used for TUNEL staining. Sections were incubated with 50 μl of equilibration buffer for 30 s, and coverslipped after the hydration step. Terminal deoxynucleotidyl transferase (Tdt, 25 μl, Tdt: reaction buffer = 1:4) was added to each section, coverslipped and incubated for 1 h at 37°C. Negative controls were incubated with water instead of Tdt. The coverslip was then removed and sections inserted in stop reaction buffer for 10 min before incubation in anti-digoxigenin-peroxidase for 40 min at room temperature, followed by DAB for color development, counterstaining in hematoxylin and coverslipping.

For both active caspase-3 and TUNEL, three sections (70 μm apart from each other) at ∼bregma -1 mm were examined. The number of positive stained neurons was manually counted in the cerebral cortex, hippocampus, and hypothalamus of the ipsilateral hemisphere. Imaging was conducted using an Olympus BX-51 light microscope (Olympus, Tokyo, Japan) with a 20× objective. Positive neuron density was presented as the percentage of positively stained neurons (brown) among total number of cells counterstained by hematoxylin (blue).

### MitoTracker Orange Staining

Mitochondrial density was evaluated by labeling formalin-fixed, paraffin-embedded tissue sections with MitoTracker Orange dye (Thermo Fisher Scientific, CA, United States). After deparaffinization and rehydration through graded ethanol solutions, the sections were incubated with MitoTracker Orange (1:5000 in PBS) for 30 min at room temperature. The sections were then rinsed with PBS for three times, cover-slipped with Vectashield mounting medium (Vector Laboratories, CA, United States), and examined by fluorescence microscopy using a fluorescein filter. ImageJ software (National Institutes of Health, Bethesda, MD, United States) was used to quantify the density of the MitoTracker staining.

### Statistical Analysis

The results are expressed as mean ± SEM. The differences between groups were analyzed using two-way ANOVA followed by Bonferroni *post hoc* tests or *t*-tests were used. *P* < 0.05 was considered significant. Prism 7.0 (GraphPad, United States) was used for statistical analysis.

## Results

### Anthropometric Parameters

To determine potential developmental retardation, body weight and whole brain weight were measured. At P45, body weight and brain weight of SE offspring were significantly smaller than the SHAM offspring (*P* < 0.01, **Table 1**). However, the percentage of brain weight standardized by body weight was not different between the groups. HI injury did not significantly affect the anthropometric parameters of the littermates (**Table 1**). Thus, maternal SE slowed down overall postnatal growth.

**Table 1:** Anthropometric parameters of the male offspring at P45.

Offspring	SHAM	HI	SE	SEHI
	*n* = 12	*n* = 12	*n* = 12	*n* = 12
Body weight (g)	20.2 ± 0.2	20.0 ± 0.3	19.1 ± 0.3^∗∗^	19.3 ± 0.3
Brain (mg)	31.1 ± 0.8	30.1 ± 0.2	29.3 ± 0.3^∗^	29.0 ± 0.3
Brain% of body weight	1.54 ± 0.04	1.51 ± 0.02	1.53 ± 0.03	1.50 ± 0.02

*Results are expressed as mean ± SEM. Data were analyzed by two-way ANOVA followed by Bonferroni *post hoc* tests. ^∗^*P* < 0.05; ^∗∗^*P* < 0.01, compared with the SHAM offspring.*

**FIGURE 1:**
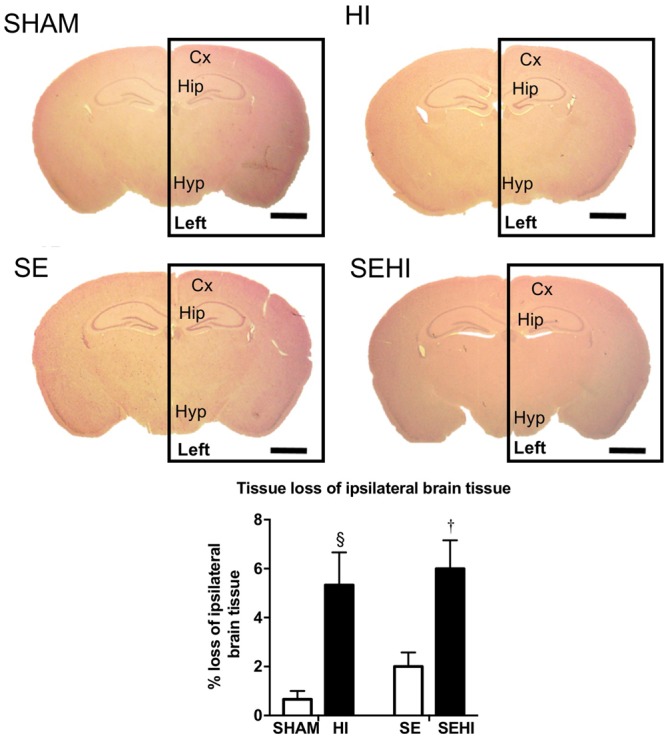
Representative images and quantification of brain size at bregma –1 mm in male offspring at P45 (*n* = 5). Scale bar = 80 μm. Results are expressed as the percentage loss of ipsilateral brain hemisphere (black rectangle enclosed area) ± SEM. *P* < 0.05 by *t*-test, ^§^ HI vs SHAM; ^†^EHI vs SE. Cx, cerebral cortex; HI, hypoxic-ischemic injury; Hip, hippocampus; Hyp, hypothalamus; SE, maternal smoke exposure; SEHI, maternal smoke exposure with hypoxic-ischemic injury.

The size of left and right hemisphere was compared to determine the impact of HI injury on brain development. Maternal SE did not significantly affect the size of the left hemisphere in non-injured littermates (SE vs SHAM, **Figure 1**). However, HI injury reduced the percentage loss of tissue of the left hemisphere in HI and SEHI offspring by 5.37 and 6.54%, respectively compared to non-injured littermates (both *P* < 0.05, **Figure 1**). The percentage loss of tissue in the left hemisphere was not significantly different between HI and SEHI offspring. Thus, HI reduced the size of the injured (ipsilateral) hemisphere, and this was not affected by maternal SE.

### Behavioral Tests

**FIGURE 2:**
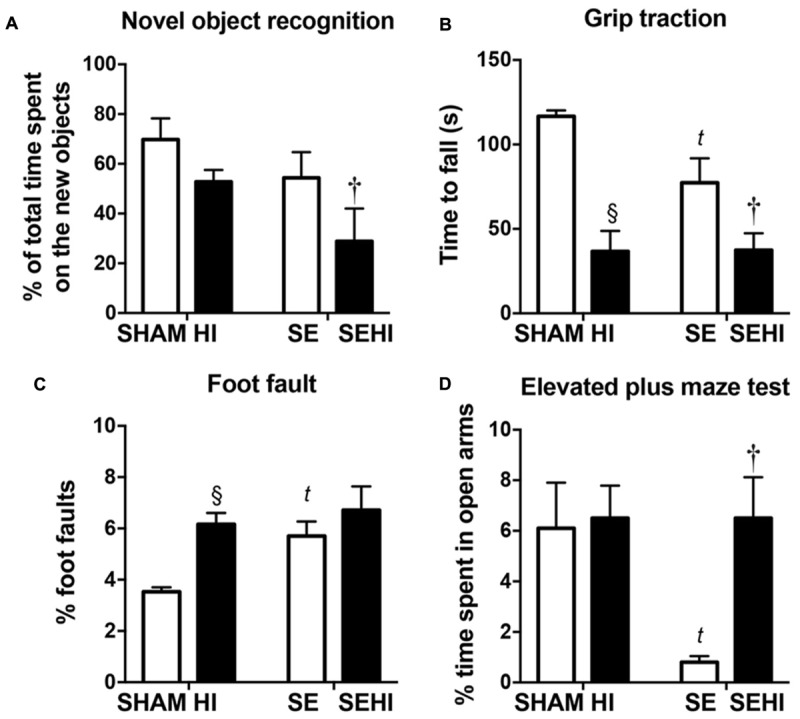
The results of novel object recognition test **(A)**, grip traction test **(B)**, foot fault test **(C)**, and elevated plus maze test **(D)** in male offspring at P40–44 (*n* = 12). Results are expressed as mean ± SEM. *P* < 0.05 by *t*-test, ^§^ HI vs SHAM; *^t^*SE vs SHAM; ^†^SEHI vs SE. HI, hypoxic-ischemic injury; SE, maternal smoke exposure; SEHI, maternal smoke exposure with hypoxic-ischemic injury.

Short-term memory was assessed using the novel objective recognition test. In this test, a mouse is expected to spend a higher percentage of time with the new object compared to the familiar object. Maternal SE led to 15% reduction of time spent with the novel object (∼50% of total time) by SE offspring compared with those from the SHAM dams (SE vs SHAM, **Figure 2A**) suggesting they were unable to recognize the familiar object. HI offspring spent 8% less time exploring the novel object (∼50% of total time) than the SHAM offspring, whereas SEHI offspring spent 50% less time interacting with the novel object than the non-injured littermates (*P* = 0.016, *F* = 1.98 vs SE, **Figure 2A**). Thus, HI injury caused a short-term memory which was more pronounced with maternal SE.

Motor function was assessed by using the grip traction and foot fault tests. In the grip traction test, SE offspring spent less time holding onto the rod compared to SHAM (*P* < 0.01, *F* = 18.2 vs SHAM, **Figure 2B**). Offspring with HI injury spent less time on the rod compared to their uninjured littermates, regardless of the maternal group (*P* < 0.0001, *F* = 12.7, SHAM vs HI; *P* = 0.018, *F* = 2.10, SE vs SEHI, **Figure 2B**). SE offspring made more foot fault errors in the foot fault test, even without injury (*P* = 0.0008, *F* = 10.8 vs SHAM, **Figure 2C**). HI offspring had a significantly increased number of foot faults (*P* < 0.0001, *F* = 6.56 vs SHAM, **Figure 2C**), but the SEHI offspring were not different compared to their non-injured littermates (**Figure 2C**). Thus, maternal SE affected forelimb muscle strength and motor coordination in the offspring, while HI further impaired muscle strength regardless of maternal group, but only impaired coordination in SHAM offspring.

Anxiety was assessed by using the elevated plus maze test. SE offspring spent 87% less time in the open arm compared to the SHAM offspring (*P* = 0.0047, *F* = 52.3 vs SE, **Figure 2D**). HI injury did not affect the anxiety level in the HI offspring, however, it normalized the anxiety level in the SEHI offspring as indicated by the time spent in the open arms (*P* = 0.0014, *F* = 42.2, SEHI vs SE, **Figure 2D**). Thus, maternal SE increased anxiety in SE offspring, which was normalized by HI injury.

### Brain Inflammatory and Oxidative Stress Markers

**FIGURE 3:**
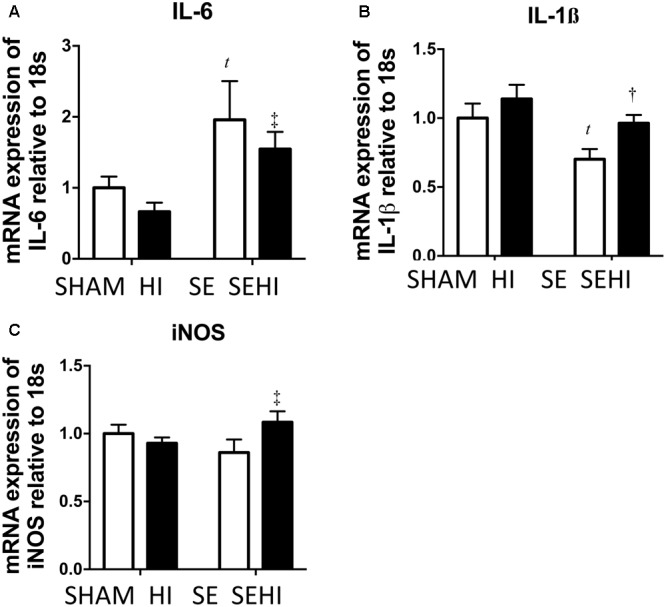
Brain mRNA expression of inflammatory markers IL-6 **(A)**, IL-1β **(B)**, and oxidative stress marker iNOS **(C)** in the male offspring at P45 (*n* = 6). Results are expressed as mean ± SEM. *P* < 0.05 by *t*-test, *^t^*SE vs SHAM; ^†^SEHI vs SE; ^‡^SEHI vs HI. HI, hypoxic-ischemic injury; SE, maternal smoke exposure; SEHI, maternal smoke exposure with hypoxic-ischemic injury; IL-6, interleukin-6; IL-1β, interleukin-1β; iNOS, inducible nitric oxide synthase.

Inflammatory responses were measured by mRNA expression of pro-inflammatory cytokines IL-6 and IL-1β and oxidative stress was measured by iNOS expression. Maternal SE increased IL-6 mRNA expression (*P* = 0.035, *F* = 12.2, SE vs SHAM, **Figure 3A**). Brain mRNA expression of IL-1β was significantly reduced by maternal SE (*P* = 0.025, *F* = 2.1, **Figure 3B**). Although HI did not further increase IL-6 mRNA expression in the SEHI group, its level was still significantly higher than the HI group (*P* = 0.006, *F* = 3.6, SEHI vs HI, **Figure 3A**). IL-1β mRNA expression was increased by HI injury in the SEHI group only (*P* = 0.012, *F* = 1.52, SEHI vs SE, **Figure 3B**). Maternal SE did not affect iNOS mRNA expression at P45 (**Figure 3C**), whereas HI injury only increased iNOS mRNA expression in the SEHI group (*P* = 0.05, *F* = 3.5, SEHI vs SE, **Figure 3C**). Thus, maternal SE increased brain inflammatory response, while HI only increased both inflammatory response and oxidative stress markers in the SEHI offspring.

### Brain Autophagy and Mitophagy Markers

**FIGURE 4:**
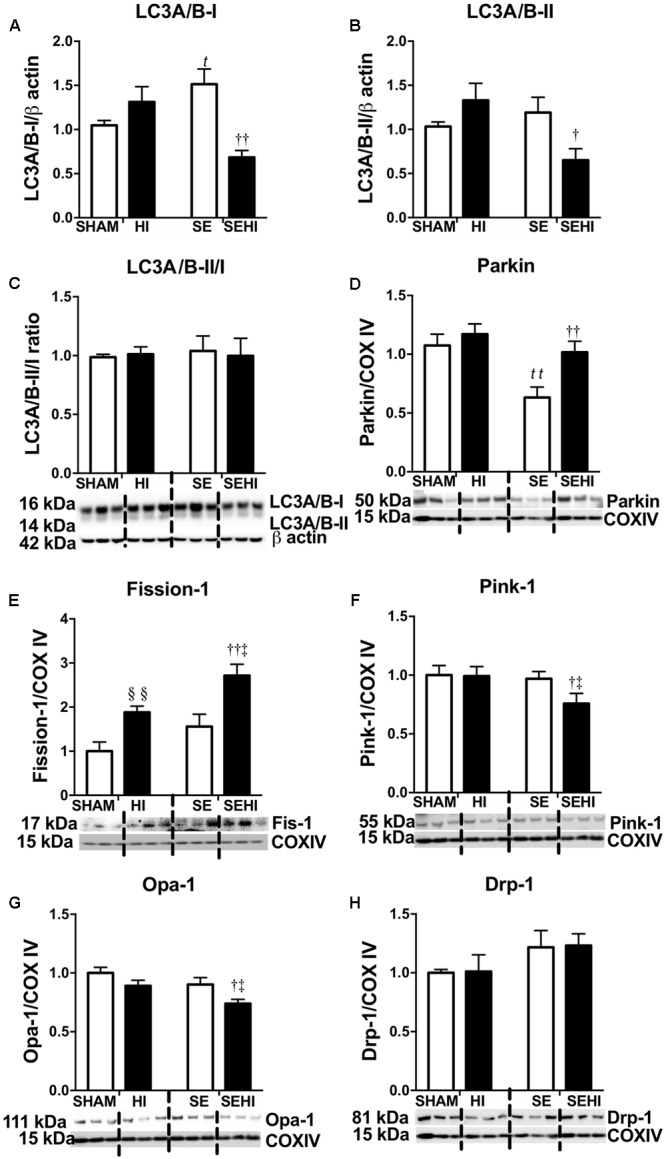
Brain protein levels of LC3A/B-I and LC3A/B-II, and LC3A/B-II/I ratio **(A–C)**, and brain mitochondrial levels of Parkin, Fission-1, Pink-1, Opa-1, and Drp-1 **(D–H)** in the male offspring at P45 (*n* = 6). Results are expressed as mean ± SEM. By *t*-test, ^§§^ *P* < 0.01, HI vs SHAM; *^t^P* < 0.05; *^tt^P* < 0.01, SE vs SHAM; ^††^*P* < 0.01; ^†^*P* < 0.05, SEHI vs SE; ^‡^*P* < 0.05, SEHI vs HI. Drp-1, dynamin-related protein-1; Pink-1, phosphatase and tensin homolog (PTEN)-induced putative kinase-1; Opa-1, optic atrophy-1; LC3A/B, light chain 3 microtubule-associated protein A/B; COX IV, cytochrome c oxidase; HI, hypoxic-ischemic injury; SE, maternal smoke exposure; SEHI, maternal smoke exposure with hypoxic-ischemic injury.

Mitochondrial integrity was assessed by autophagy and mitophagy markers. Maternal SE increased LC3A/B-I protein levels in the SE offspring (*P* = 0.03, *F* = 9.54, SE vs SHAM, **Figure 4A**). Maternal SE also decreased Parkin level in the offspring (*P* = 0.0048, *F* = 1.149, SHAM vs SE, **Figure 4D**). HI injury reduced LC3A/B-I (*P* = 0.002, *F* = 4.77, SEHI vs SE, **Figure 4A**) and LC3A/B-II (*P* = 0.047, *F* = 1.24, SEHI vs SE, **Figure 4B**) protein levels in the SEHI group only. However, Fission-1 levels in both HI and SEHI groups were significantly increased by HI injury compared with their non-injured littermates (*P* = 0.0038, *F* = 2.22, HI vs SHAM; *P* = 0.0081, *F* = 1.18, SEHI vs HI, **Figure 4E**), while it only increased Parkin in the SEHI group (*P* = 0.0088, *F* = 1.099 vs SE, **Figure 4D**). HI injury reduced Pink-1 (*P* = 0.036, *F* = 1.98 vs SE, **Figure 4F**) and Opa-1 protein levels only in the SEHI group (*P* = 0.02, *F* = 2.67 vs SE, **Figure 4G**). Drp-1 was not affected in both HI and SEHI groups (**Figure 4H**). Overall, autophagy may not be affected by maternal SE, but fission activity may be increased in the SE offspring to break down damaged mitochondria. HI increased fission marker, but reduced autophagy and fusion markers in the SEHI offspring only.

### Brain Markers of Mitochondrial Function

**FIGURE 5:**
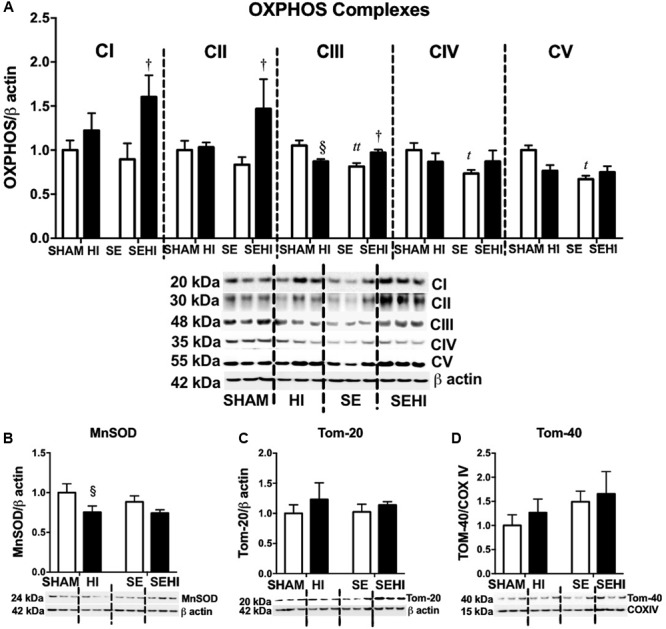
Brain mitochondrial protein levels of OXPHOS complexes I–V, MnSOD, Tom-20, and Tom-40. **(A–D)** in male offspring at P45 (*n* = 6). Results are expressed as mean ± SEM. By *t*-test, ^t^*P* < 0.05; ^tt^*P* < 0.01, SE vs SHAM; ^§^*P* < 0.05, HI vs SHAM; ^†^SEHI vs SE. MnSOD, manganese superoxide dismutase; Tom, translocase of the mitochondrial outer membrane; OXPHOS, oxidative phosphorylation; COX IV, cytochrome c oxidase; HI, hypoxic-ischemic injury; SE, maternal smoke exposure; SEHI, maternal smoke exposure with hypoxic-ischemic injury.

OXPHOS, MnSOD, and Tom proteins were measured as the indicators of mitochondrial function. Maternal SE reduced brain OXPHOS complexes III–V protein levels (CIII: *P* = 0.001, *F* = 2.07; CIV: *P* = 0.02, *F* = 3.92; CV: *P* = 0.02, *F* = 4.39 vs SHAM, **Figure 5A**). HI injury significantly reduced MnSOD (*P* = 0.04, *F* = 1.65 vs SHAM, **Figure 5B**) and OXPHOS complex III levels in the offspring from the HI dams only (*P* = 0.02, *F* = 4.66 vs SHAM, **Figure 5A**). In contrast, the levels of OXPHOS complexes I–III were increased in SEHI offspring (CI: *P* = 0.04, *F* = 1.82; CII: *P* = 0.04, *F* = 15; CIII: *P* = 0.02, *F* = 1.25 vs SE, **Figure 5A**). Tom-20 and Tom-40 levels were not affected by maternal SE (**Figures 5C,D**), nor HI injury (**Figures 5C,D**). Thus, maternal SE suppressed OXPHOS functional units in the offspring, while HI injury only reduced MnSOD level in the HI offspring.

### Brain Mitochondrial Density

**FIGURE 6:**
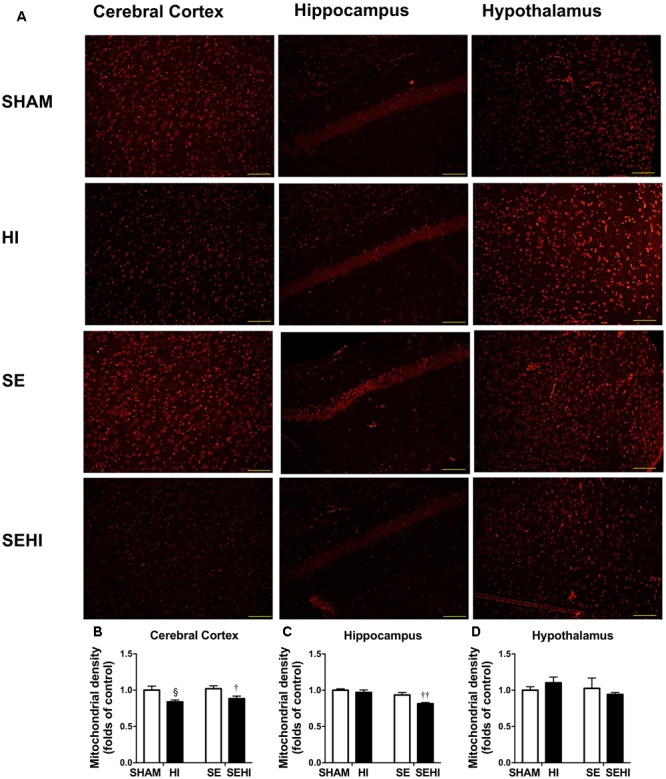
Mitochondrial density in the cerebral cortex, hippocampus, and hypothalamus in the male offspring at P45 (*n* = 5) **(A–D)**. Results are expressed as mean ± SEM. By *t*-test, ^§^ *P* < 0.05, HI vs SHAM; ^††^*P* < 0.01; ^†^*P* < 0.05, SEHI vs SE. Scale bar = 40 μm. HI, hypoxic-ischemic injury; SE, maternal smoke exposure; SEHI, maternal smoke exposure with hypoxic-ischemic injury.

Mitochondria were stained with MitoTracker Orange (shown as orange fluorescence) against the dark background in the images (**Figure 6**). Maternal SE did not affect mitochondrial density in the cerebral cortex, hippocampus, and hypothalamus. However, HI injury reduced mitochondrial density in the cerebral cortex on both HI and SEHI offspring compared with their non-injured littermates (*P* = 0.02, *F* = 6.63, HI vs SHAM; *P* = 0.02, *F* = 1.40, SEHI vs HI, **Figure 6B**). Mitochondrial density in the hippocampus was also reduced in the SEHI offspring (*P* = 0.008, *F* = 3.80 vs SE, **Figure 6C**). There was no change in mitochondrial density in the hypothalamus (**Figure 6D**). Thus, mitochondrial density was significantly reduced by HI injury, especially in the cortex.

### Apoptotic Markers

**FIGURE 7:**
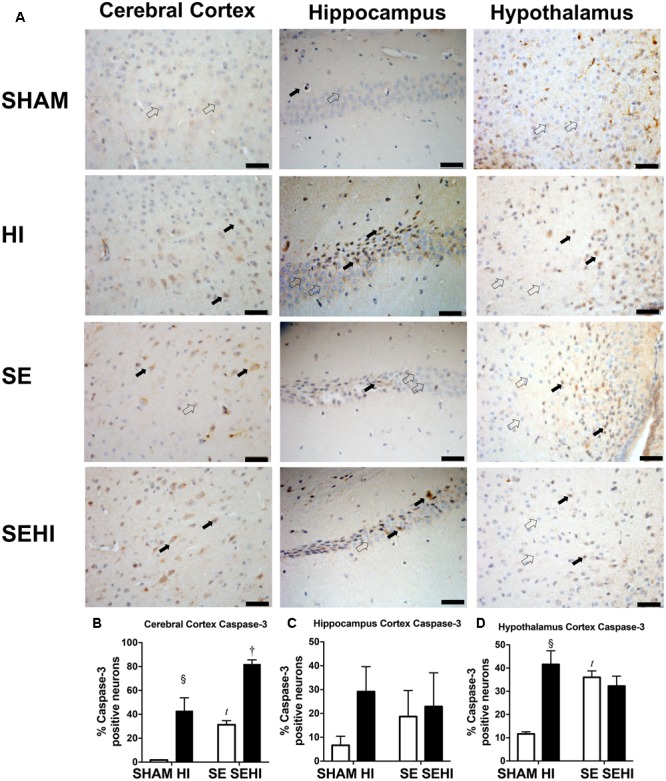
Immunostaining of active caspase-3 in cerebral cortex, hippocampus, and hypothalamus in the male offspring at P45 (*n* = 5) **(A–D)**. Caspase-3 positive (closed arrow) and caspase-3 negative (open arrow). Scale bar = 20 μm. Results were expressed as mean ± SEM. *P* < 0.05 by *t*-test, *^t^*SE vs SHAM; ^§^ HI vs SHAM; ^†^SEHI vs SE. HI, hypoxic-ischemic injury; SE, maternal smoke exposure; SEHI, maternal smoke exposure with hypoxic-ischemic injury.

Apoptosis was assessed by caspase-3 and TUNEL staining. Maternal SE increased the percentage of active caspase-3 positive cells in the cerebral cortex (*P* = 0.0001, *F* = 183.43 vs SHAM offspring, **Figure 7B**) and hypothalamus in the offspring (*P* = 0.0001, *F* = 6.44, SE vs SHAM offspring, **Figure 7D**). HI injury increased the percentage of active caspase-3 positive cells in the cerebral cortex in both HI and SEHI offspring (*P* = 0.005, *F* = 1947, HI vs SHAM; *P* = 0.0002, *F* = 1.12, SEHI vs HI, **Figure 7B**). Its change in the hippocampus was not statistically significant (**Figure 7C**). HI injury also increased the percentage of active caspase-3 positive cells in the hypothalamus, however, only in the HI offspring (*P* = 0.001, *F* = 39.5, HI vs SHAM, **Figure 7D**).

**FIGURE 8:**
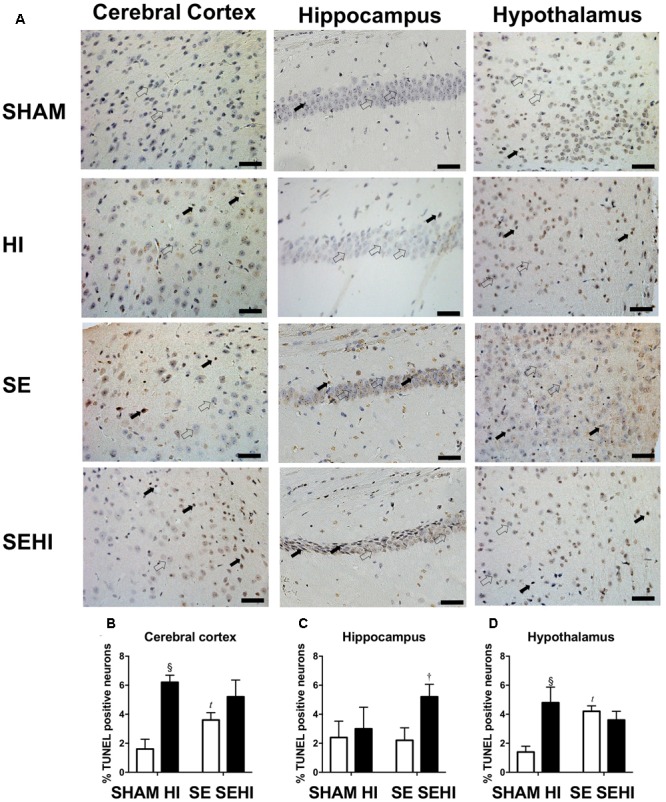
TUNEL staining in cerebral cortex, hippocampus, and hypothalamus in the male offspring at P45 (*n* = 5) **(A–D)**. TUNEL positive (closed arrow) and TUNEL negative (open arrow). Scale bar = 20 μm. Results are expressed as mean % TUNEL positive neurons ± SEM. By *t*-test; *^t^P* < 0.05, SE vs SHAM; ^§^*P* < 0.01, HI vs SHAM; ^†^*P* < 0.05, SEHI vs SE. HI, hypoxic-ischemic injury; SE, maternal smoke exposure; SEHI, maternal smoke exposure with hypoxic-ischemic injury.

Maternal SE increased the percentage of TUNEL positive cells in the cerebral cortex (*P* = 0.02, *F* = 1.77, SE vs SHAM, **Figure 8B**) and hypothalamus (*P* = 0.009, *F* = 1.422, SE vs SHAM, **Figure 8D**) in SE offspring. HI injury increased the percentage of TUNEL positive cells in the cerebral cortex (*P* = 0.0003, *F* = 1.92, HI vs SHAM, **Figure 8B**) and hypothalamus in the offspring from SHAM exposed dams (*P* = 0.003, *F* = 4.36, HI vs SHAM, **Figure 8D**). There was also an increase in the percentage of TUNEL positive cells in the hippocampus in the SEHI offspring compared to their non-injured littermates (*P* = 0.02, *F* = 1.0, SEHI vs SE, **Figure 8**). Thus, HI injury is a more potent factor than maternal SE to increase brain apoptosis in multiple brain regions.

## Discussion

In this study, even without postnatal HI injury, SE offspring displayed both motor and cognitive dysfunction, as has been previously observed in human studies (Knopik, 2009). With the early postnatal HI injury, maternal SE did not significantly worsen most of the functional deficits except for short-term memory function. However, it did exacerbate some of the cellular abnormalities observed in the brain after HI injury, including increased apoptosis in the cortex and enhanced DNA fragmentation and reduced mitochondrial density in the hippocampus.

### Effect of Maternal Cigarette Smoke Exposure

Maternal smoking can increase the risk of placental infarction and microinfarctions (Kaminsky et al., 2007), which can interrupt blood supply to parts of the placenta. This induces placental ischemia to reduce blood and oxygen supply to the fetus causing brain underdevelopment (Fatemi et al., 2009). Nicotine can also be transmitted through the breast milk to offspring to directly affect the growth (Chan et al., 2016b). As such, SE offspring had reduced body weight and brain weight which was consistent with our previous study (Chan et al., 2016b).

Maternal SE reduced forelimb grip strength and increased the number of foot faults in offspring even without HI injury, which may be directly linked to increased cortical markers of apoptosis and DNA fragmentation in these SE offspring. The grip test and foot fault tests can be used to objectively demonstrate deficits in motor function (Rogers et al., 1997). Our results are consistent with different retrospective studies in humans which correlated maternal smoking to impaired locomotor function in offspring. A study in Denmark, Norway, and Sweden indicated that maternal smoking has a weak correlation with balance in 5-year-old children (Trasti et al., 1999). On the other hand, maternal smoking tends to reduce motor competence in 11 years old children, particularly on the non-dominant side (Larsson and Montgomery, 2011). The SE offspring displayed an increase in anxiety as reflected by less time spent in the open arms during the elevated plus maze test due to fear of the open environment. This is in line with a previous animal study which showed that prenatal nicotine exposure can increase anxiety levels (Moylan et al., 2015). In rats, nicotine administration can lead to an anxiogenic effect in a dose-dependent manner (Cheeta et al., 2001). The anxiety-producing effect is more striking in adolescent female rats with low doses of nicotine (0.05 and 0.10 mg/kg) and in adolescent males with a high dose of nicotine (0.25 mg/kg) (Cheeta et al., 2001). Maternal smoking during pregnancy is also a risk factor for antisocial behavior, anxiety, and depression in both male and female offspring in humans into adolescence and adult (Fergusson et al., 1998; Brennan et al., 1999). Deficits in motor coordination have been shown to be mediated by increased IL-6 levels in mice with mild traumatic brain injury (Yang et al., 2013). Thus, here, the motor deficits observed in SE offspring may be mediated through upregulated brain IL-6 mRNA expression. Taken together, these studies along with our own data indicate that maternal SE leads to some baseline motor and cognitive changes in offspring that can be observed into adolescence.

Autophagy is a process where cells digest proteins, lipids, and organelles in the cytoplasm, for removal and turnover (Ashrafi and Schwarz, 2013). Autophagy can be examined through assessing LC3A/B proteins. The process of autophagy in mitochondria is called mitophagy (Ashrafi and Schwarz, 2013) which can be further divided into fusion and fission (Bereiter-Hahn, 1990; Westermann, 2010). The fragmentation of damaged mitochondria is facilitated by fission proteins Drp-1 and Fission-1 (Onoue et al., 2013) which slice the inner and outer mitochondrial membranes, respectively (Elgass et al., 2013). Healthy portions of the mitochondria are combined through the fusion process facilitated by Opa-1 proteins (Kanazawa et al., 2008). Mitochondrial fragmentation is associated with increased neuronal cell death (Wang et al., 2013). Among different proteins associated with mitophagy, Parkin labels damaged mitochondria for autophagic degradation (Narendra et al., 2008). Maternal SE has been linked to reduced Parkin protein levels which may compromise the removal of damaged mitochondria (Wang et al., 2013). Reduced Parkin was found to be related to changes in mitochondrial morphology and neuronal apoptosis (Wang et al., 2013). In the current study, there was an increase in the apoptotic marker active caspase-3 in the cerebral cortex of SE offspring. LC3A/B-I protein levels were increased in SE offspring, but its conversion to LC3A/B-II remained unchanged. This may indicate that damaged mitochondria may not be able to be engulfed by the autophagosome for recycling. As a result, while maternal SE caused increased mitochondrial fission and reduced fusion in brain tissue, it did not lead to a significant change in the mitochondrial density in the brain regions examined in this study. This may be due to the capacity of the MitoTracker staining method, which cannot distinguish between healthy and damaged mitochondria. Mitochondria are not well protected from oxidative stress as there is limited resource of the endogenous antioxidant enzyme MnSOD (Holley et al., 2011). Once mitochondria are damaged, the production of antioxidants is also reduced. We have observed increased oxidative cell damage in the brains of SE offspring in our previous study (Chan et al., 2016b). Reduced OXPHOS complexes III–V in this study may well suggest impaired mitochondrial function, although the mitochondrial density itself was unchanged by maternal SE.

Smoking can affect the functions of multiple organ system, including the lung, brain, heart, and kidney. It is clear from this and our previous studies, such impact can be transferred to the next generation, including that of nicotine addiction and substance abuse (Yohn et al., 2015; Chan et al., 2016b; Vivekanandarajah et al., 2016; Yuan et al., 2016; Sukjamnong et al., 2017). The programming in offspring can also be long-lasting as shown in other studies, especially the impact on neurocognitive changes such as increased anxiety-like behavior in this study (Mueller and Bale, 2007, 2008; Vassoler and Sadri-Vakili, 2014; Bale, 2015). Such transgenerational effects have been suggested to be mediated through epigenetic modifications (Yohn et al., 2015), where brain-derived neurotrophic factor has been proposed as the key player in cognitive disorders (Vassoler and Sadri-Vakili, 2014). To mitigate the adverse effect of maternal SE on neurological outcomes in offspring, quitting smoking prior to the pregnancy is desired. Combatting nicotine addiction remains a challenge for smokers, where nicotine replacement therapy does not always yield high success rate of quitting smoking (Osadchy et al., 2009). Exercise may reduce nicotine craving (Sanchez et al., 2015), while new treatment developments such as deep brain stimulation, repeated transcranial magnetic stimulation, or novel drug targets may be new options to facilitate better smoking cessation prior to pregnancy (Kravitz et al., 2015; Shen et al., 2016; Ubaldi et al., 2016; Wang et al., 2016).

### Effect of Hypoxic-Ischemic Injury

In this study, HI injury did not affect the brain weight in the offspring from the SHAM dams, but it reduced the size of the hemisphere ipsilateral to the injury. In this study, the HI injury was milder compared to a study by Li et al. (2012b) who showed that perinatal oxygen exposure increased infarct size in male Sprague-Dawley rats 48 h after HI injury. The difference between our study and Li et al. (2012b) might be due to differences in hypoxic exposure and species used. In this study, mice were exposed to low ambient oxygen levels (8% oxygen) for 30 min, whereas Li et al. (2012b), exposed rats to 8% oxygen for an extended period of 2.5 h.

HI injury in the SHAM offspring only marginally affected their short-term memory function as reflected by their performance in the novel object recognition test. This likely related to lack of injury to the regions related to this function. It needs to be noted that there was no measurable hippocampal damage in terms of apoptosis markers following HI injury. A study from Reinboth et al. (2016) showed that there was a reduction of memory function in novel object recognition test at P32 which recovered on P46 in mice with HI injury. Our result is somewhat consistent with this study where the decline in short-term memory function was not remarkable at P40–44, suggesting that neural plasticity is more resilient to neonatal HI injury. HI injury regardless of its severity, was found to reduce anxiety-like behavior in Sprague-Dawley rats due to decreased activity of tyrosine hydroxylase in the substantia nigra (Hei et al., 2012). However, in the current study, HI injury had no effect on the anxiety levels in HI offspring. HI injury significantly impaired forelimb grip strength and increased mistakes during walking. This aligns with the reduction in mitochondrial density and the increase in apoptotic markers in the cerebral cortex. The observation in the current study is similar to what has been shown in mice following mild traumatic brain injury (Yang et al., 2013).

One of the early responses to HI injury in human newborns is an increase in brain IL-1β level (Savard et al., 2015) which may also be attributed to increased release from microglia, astrocytes, and neurons (Liu and McCullough, 2013). Unexpectedly, we did not see an increase in IL-1β in HI mice. Yet, it cannot be excluded as a possibility that we did not differentiate amongst the brain regions with evidence showing the changes in IL-1β in relation to the injury can be brain region, time and duration of hypoxia exposure dependent (Eriksson et al., 1999; Albertsson et al., 2014). Nevertheless, the markers of apoptosis and DNA damage were significantly increased following HI injury in cerebral cortex and hypothalamus, in line with the decline in behavioral function observed in HI treated mice.

During HI, there are substantial increases in energy needs in the brain, and mitochondria play a vital role in post-injury brain adaptations. However, this can result in an increase in free radicals as a byproduct during ATP synthesis and can further damage the mitochondria, especially when the endogenous MnSOD is insufficient to counteract increased oxidative stress as shown in the brain of the HI offspring in this study. As such, the markers of mitochondrial fission are increased in brains of the HI offspring, as mitochondrial fission aids in the removal of damaged mitochondrial portions for degradation (Onoue et al., 2013).

### The Interaction between Maternal Cigarette Smoke Exposure and Hypoxic-Ischemic Injury

In this study, maternal SE did not exacerbate the extent of brain injury. On the other hand, HI injury normalized the anxiety level in the SEHI offspring, perhaps due to a higher baseline anxiety level in non-injured SE offspring. Maternal SE did not induce any further motor deficits in the forelimb grip strength test suggesting that HI injury is a more potent stressor than maternal SE to produce a deficit in this function. The foot fault errors made in the grid walking test were not changed in the SEHI group compared with their non-injured SE littermates who already showed motor incoordination in relation to control. This suggests the resilience of the brain to maintain basic coordination function in SEHI offspring compared to SE offspring.

SEHI offspring also had a reduction of ability to recognize novel objects when compared with HI offspring, which is consistent with the increase in DNA fragmentation in the hippocampus. SEHI offspring spends 30% of time on the new object, indicating they were unable to process spatial information (Akar et al., 2014). This may be due to reduced anxiety level compared with non-injured littermates. Such finding is consistent with the literature where anxiolytic agents such as 7-nitroindazole and 1H-[1,2,4]oxadiazolo[4,3-1] quinoxaline-1-one reduce percentage of time spent on novel object with reduced anxiety level in the mice (Akar et al., 2014). Thus, maternal SE interacts with HI to impair cognitive functional outcomes in offspring in adolescences (Akar et al., 2014).

IL-1β mRNA was increased in SEHI offspring compared with the non-injured littermates, and this was associated with increased levels of iNOS and markers of cell apoptosis and DNA fragmentation in the hippocampus. Increased levels of IL-1β in umbilical cord blood correlate with enhanced severity of HI injury in human infants affected by maternal SE (Liu and Feng, 2010). This indicates that maternal SE increased cellular injury after HI injury most likely due to increased oxidative stress during gestation.

Fission-1 levels were higher in SEHI offspring with brain injury suggesting that HI injury caused more severe mitochondrial damage in SEHI offspring. Both Parkin and Pink-1 function to promote mitochondrial fission through Drp-1. Pink-1 is also recruited to depolarized mitochondria and phosphorylates Parkin to ubiquitinate and degrade damaged mitochondria (Kim et al., 2008; Shiba-Fukushima et al., 2012). Pink-1 has been shown to protect different cell types from various stressors, including oxidative stress, mitochondrial blockers and apoptosis (Valente et al., 2009). In the current study, Pink-1 levels were reduced by the interaction between maternal SE and HI injury, suggesting compromised neuroprotective function in SE offspring following HI injury. Pink-1 also initiates the translocation of Parkin onto damaged mitochondria; however, the increase in Parkin and Fission-1 may still activate the fission process without Pink-1 (Dagda et al., 2009), which can reduce autophagosomes as shown in SEHI offspring. This may further compromise autophagy leading to cell death (Kouno et al., 2005). Indeed, reduced LC3A/B-II and increased markers of apoptosis were observed in the cerebral cortex of the SEHI offspring in the current study. Furthermore, a reduction in Opa-1 levels in SEHI offspring also indicates that there may be less healthy mitochondrial fragments to regenerate new mitochondria due to the interaction between maternal SE and HI injury. Maternal SE did not affect mitochondrial density reduction in cerebral cortex by HI, but it only induced a reduction in the hippocampus following the injury; whereas their OXPHOS complexes I and III proteins were adaptively increased suggesting increased ATP demand for damage repair. Increased OXPHOS activity can also increase the production of free radicals (Turrens and Boveris, 1980; Sugioka et al., 1988), resulting in further increase in oxidative stress reflected by upregulated iNOS expression observed in the SEHI offspring; a level that exceeded the capacity of endogenous MnSOD to counteract. SEHI offspring had much higher levels of apoptosis and DNA damage markers than the HI offspring in selected brain regions following HI injury, in the face of increased markers of inflammation, mitochondrial fission but reduced mitochondrial fusion, suggesting more severe mitochondrial damage. This may explain why some of the neurological and cellular outcomes following HI injury were worse in the SEHI offspring than the HI offspring.

## Conclusion

Maternal SE alone impaired several neurological functions in male offspring, while the additional HI injury did not have pronounced additive effect on most of these behavioral outcomes. However, maternal SE worsened markers of brain cell apoptosis, inflammation, oxidative stress, and mitophagy in offspring following HI injury. Further study is needed to investigate their long-term impact and functional outcomes in adult SE offspring with HI brain injury in early life.

## Author Contributions

HC, NJ, and SS designed the study. YC and HC performed all the experiments, collected the data, and analyzed the data. YC, SS, RM, BO, BV, CP, NJ, and HC contributed to the writing of the main manuscript text, and YC prepared **Figures 1–8** and **Table 1**. All authors reviewed the manuscript.

## Conflict of Interest Statement

The authors declare that the research was conducted in the absence of any commercial or financial relationships that could be construed as a potential conflict of interest.
