# Erratum: Maternal Cigarette Smoke Exposure Worsens Neurological Outcomes in Adolescent Offspring with Hypoxic Ischemic Injury

**DOI:** 10.3389/fnmol.2018.00084

**Published:** 2018-03-12

**Authors:** Frontiers Production Office

**Affiliations:** Frontiers Production Office, Frontiers Media SA, Lausanne, Switzerland

**Keywords:** mitophagy, apoptosis, cognition, motor behavior

Due to a typesetting error, Figure 2 in the final pdf was incorrect. The correct version appears below.

**Figure 2:**
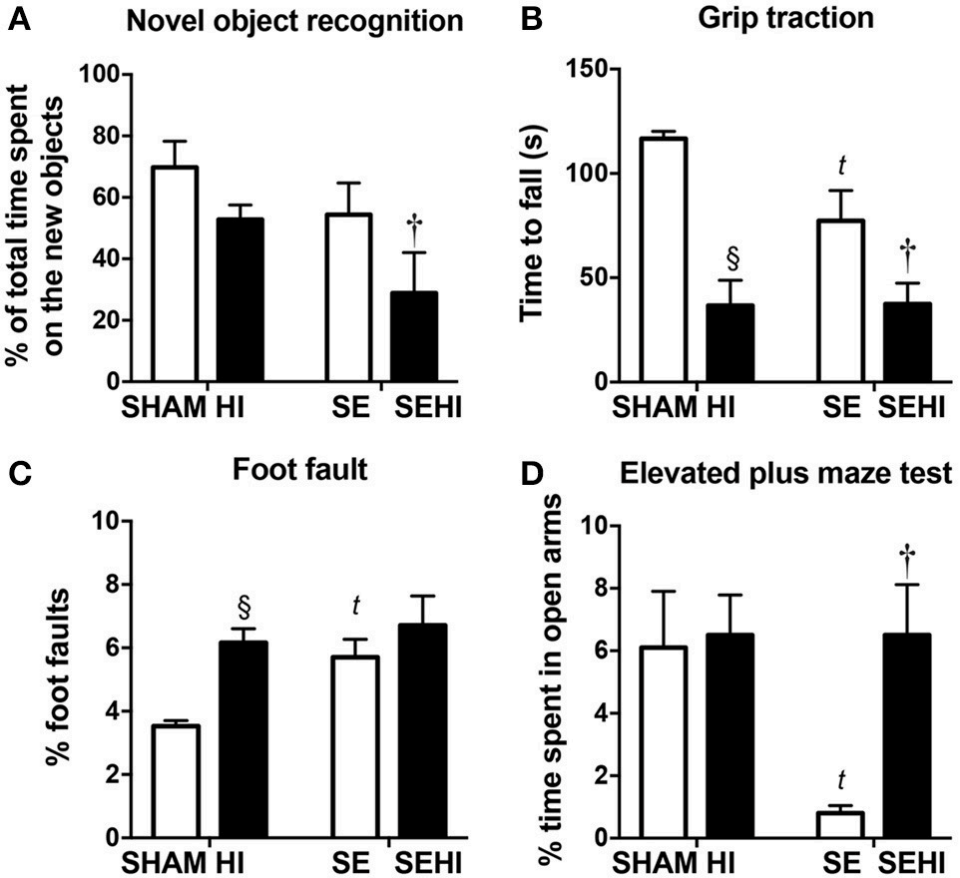
The results of novel object recognition test **(A)**, grip traction test **(B)**, foot fault test **(C)**, and elevated plus maze test **(D)** in male offspring at P40–44 (*n* = 12). Results are expressed as mean ± SEM. *P* < 0.05 by *t*-test, ^§^HI vs. SHAM; ^*t*^SE vs. SHAM; ^†^SEHI vs. SE. HI, Hypoxic-ischemic injury; SE, maternal smoke exposure; SEHI, maternal smoke exposure with hypoxic-ischemic injury.

The publisher apologizes for this error and the original article has been updated to reflect this. This error does not change the scientific conclusions of the article in any way.

